# Supplementary material for: Evolutionary rescue by compensatory mutations is constrained by genomic and environmental backgrounds
Source: Mol Syst Biol. 2015 Oct 12;11(10):832. doi: 10.15252/msb.20156444 (PMC4631203; doi:10.15252/msb.20156444)
Supplement: Supplementary file 1 — Expanded View Figures PDF [file msb0011-0832-sd1.pdf]

Expanded View Figures

```
                            1        10        20        30        40        50        60        70        80        90       100
                            |         |         |         |         |         |         |         |         |         |         |
Homo sapiens                MSGG--PMGGRPGGRGAPAVQQNIPSTLLQDHENQRLFEMLGRKCLTLATAVVQLYLALPPGAEHWTKEH-CGAVCFVKDNPQKSYFIRLYGLQAGR-LL
Mus musculus                MNSGPGPVGGRPGGRGGPAVQQNIPSNLLQDHENQRLFELLGRKCWTLATTVVQLYLALPPGAEHWTMEH-CGAVCFVKDNPQKSYFIRLYGLQAGR-LL
Canis lupus familiaris      MSGG--PVGGRSGGRGGPGVQQNIPSTLLQDHENQRLFEMLGRKCWTLATAVVQLYLALPPGAEHWTKEH-CGAVCFVKDNPQKSYFIRLYGLQAGR-LL
Rattus norvegicus           MSSG--PVGGRSGGRGGPAVQQNIPSNLLQDHENQRLFELLGRKCWTLATAVVQLYLALPPGAEHWTMEH-CGAVCFVKDNPQKSYFIRLYGLQAGR-LL
Danio rerio                 M---------RRAAKGK--AQECSGSSLLSIQENEKLEDLLGRRCVSLSSAVVQLLMALPSDPNRWTPQQ-SGVVCFVKDSSLRSFFIRLYDIKAGK-LV
Saccharomyces cerevisiae    M--------GLLNSSDKEIIKRALPKA-----SN-KIIDV----------TVARLYIAYP-DKNEWQYTGLSGALALVDDLVGNTFFLKLVDINGHRGVI
```

Figure EV1. Sequence alignment of WASP and its homologs.
The conserved W64 residue is shown in bold. Sequences were obtained from the Ensembl database and aligned with Geneious 6.1.6.

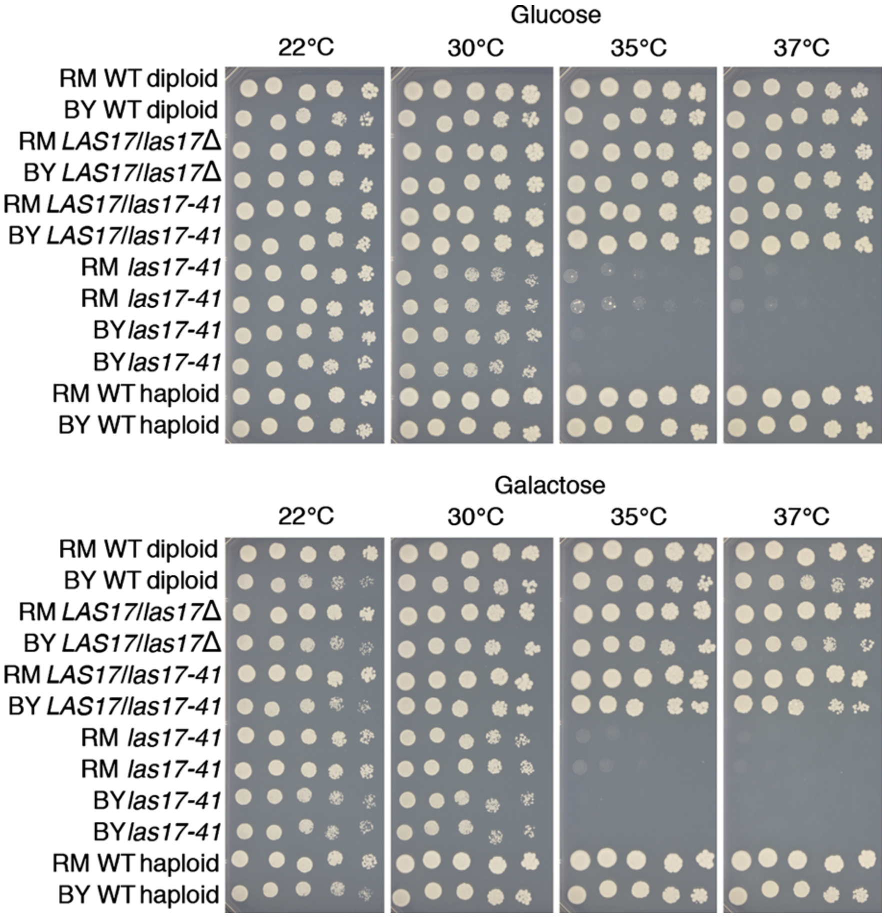

Figure EV2. The thermosensitive phenotype of *las17-41* on glucose and galactose synthetic media in BY and RM genetic background is recessive, showing a loss-of-function mutation.
The *las17-41* allele causes a comparable thermosensitive phenotype in a haploid BY and RM genetic background on both carbon sources. The mechanism causing thermosensitivity is not a gain of function, because *LAS17/las17-41* and *LAS17/las17*Δ hemizygous strains have the same growth phenotype as their wild type in both backgrounds. Spot assay shown after 3 days of growth.

© 2015 The Authors

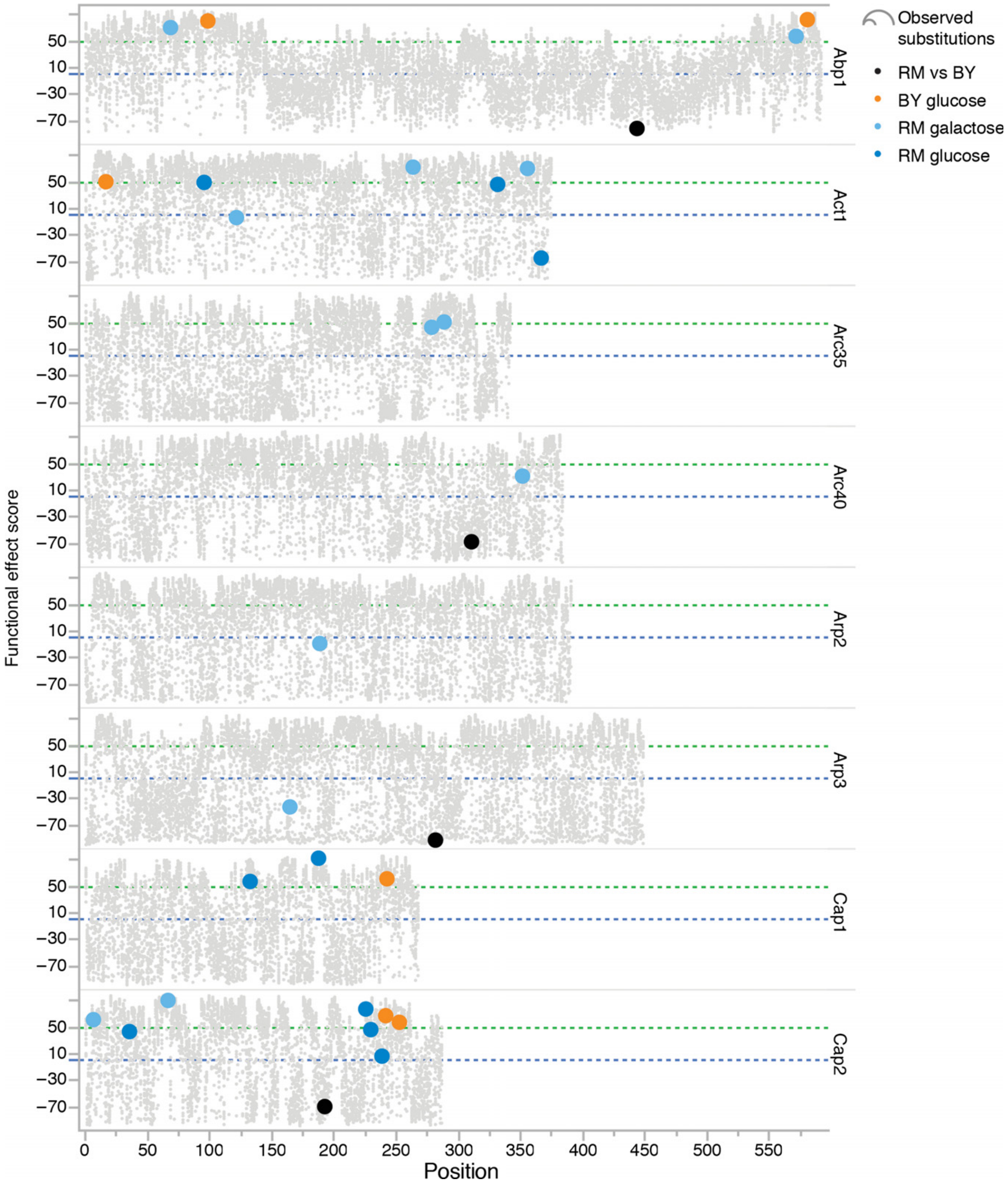

Figure EV3. Predicted functional effects of point mutations.

Functional effect scores were computed with SNAP2 (Yachdav *et al*, 2014), a neural network-based method that uses *in silico* derived protein information to make predictions regarding functionality of individual substitutions. A score > 50 indicates a strong signal for effect, −50 < score < 50 indicates a weak signal, and a score < −50, a strong signal for neutral/no effect. Each dot corresponds to a possible substitution for each position of a protein. Larger dots were substitutions observed in the experiment. Dots are colored according to the experimental context in which they were observed. Black dots indicate the pre-existing SNP between the two ancestral genomes.

 © 2015 The Authors

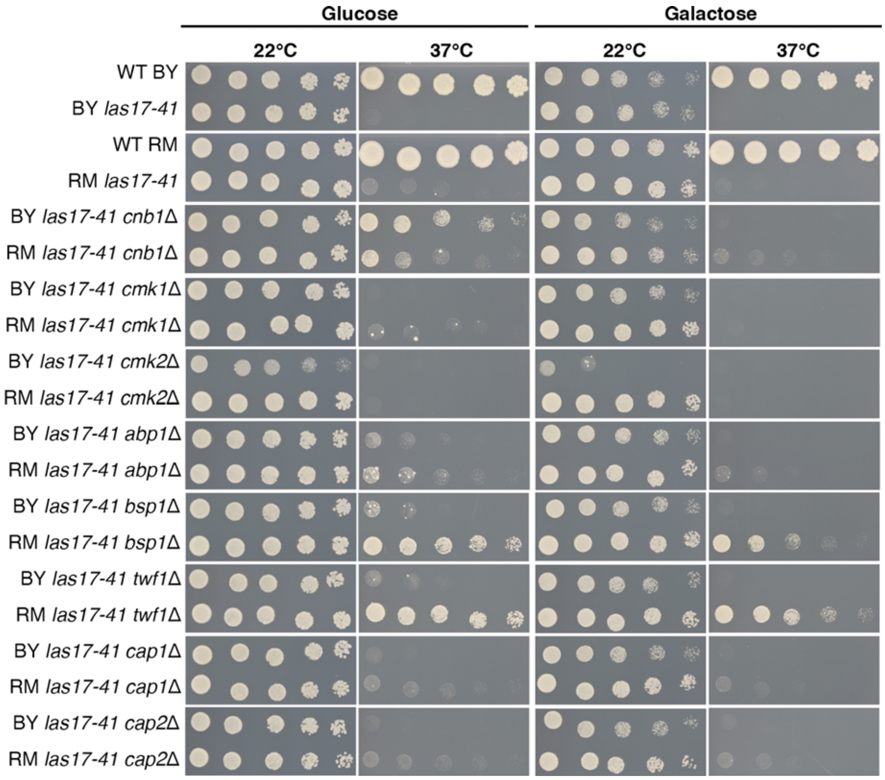

Figure EV4. Compensation by loss of function.
Spot dilution assay shown after 3 days of growth on SC glucose or galactose media for whole gene deletions of genes identified by genome sequencing in the thermosensitive strains. The results confirm that the loss of function of certain genes can counterweight *las17-41* thermosensitivity, in a context-specific manner. The case of *twf1*Δ in particular is restricted to the RM genetic background as previously observed among the sequenced mutants. The deletion of *bsp1* follows the same pattern, but spontaneous mutants can be observed in the BY background on glucose. It is noteworthy that the only BY *bsp1* mutant recovered in the experiment was also diploid; hence, *bsp1* mutations may be compensatory in this background on glucose only in combination with another mutational event.

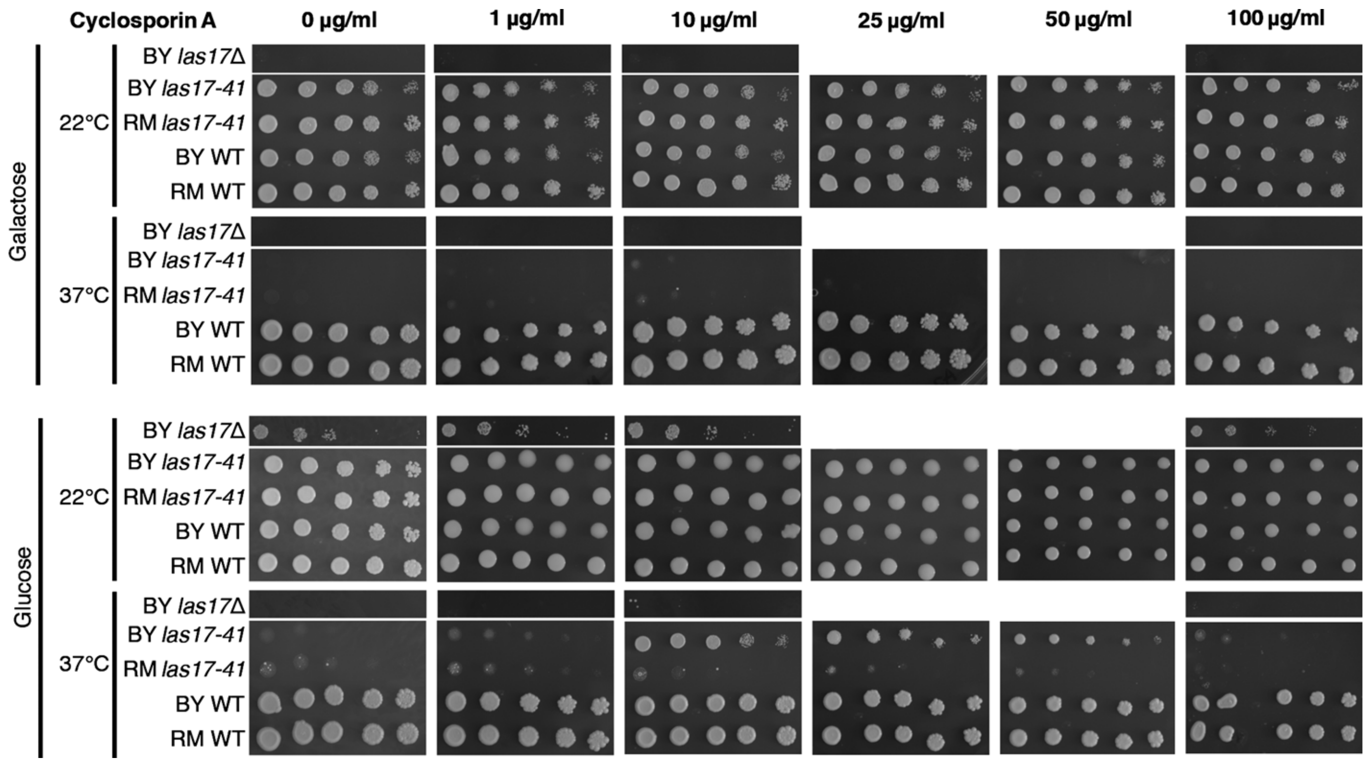

Figure EV5. Pharmacological compensation of *las17-41* thermosensitivity by cyclosporin A is optimal at 10 µg/ml.

© 2015 The Authors
